# A phase I, randomized, open-label, single-dose, 3-period crossover study to evaluate the drug-drug interaction between ZX008 (fenfluramine HCl oral solution) and a regimen of stiripentol, clobazam, and valproate in healthy subjects 

**DOI:** 10.5414/CP203276

**Published:** 2018-10-19

**Authors:** Brooks Boyd, Steven Smith, Arnold Gammaitoni, Bradley S. Galer, Gail M. Farfel

**Affiliations:** Zogenix, Inc., Emeryville, CA, USA

**Keywords:** Dravet syndrome ‒ drug interaction ‒ fenfluramine ‒ pharmacokinetics ‒ stiripentol

## Abstract

Objective: Phase I, open-label, randomized, single-dose, 3-period crossover study assessing pharmacokinetics (PK) and safety of ZX008, a liquid oral formulation of fenfluramine (FFA) under development for adjunctive treatment of Dravet syndrome and Lennox-Gastaut syndrome, administered with and without a combined antiepileptic drug (AED) regimen of stiripentol (STP), valproate (VPA), and clobazam (CLB) (STP regimen). Materials and methods: 26 healthy adults were administered the following treatments: ZX008 0.8 mg/kg; STP 3,500 mg, CLB 20 mg, VPA 25 mg/kg (max. 1,500 mg); and ZX008 0.8 mg/kg + STP regimen. Dose periods were 17 days apart. Blood samples were obtained for 72 hours after drug administration and used to calculate non-compartmental PK parameters. Results: Statistical bioequivalence-type analysis demonstrated ZX008 had no significant impact on the PK of any drug in the STP regimen, while the STP regimen moderately affected FFA PK. The 3-drug combination increased the geometric mean C_max_, AUC_0–t_, and AUC_0–inf_ of FFA while reducing the C_max_ and AUC_0–t_ of its major metabolite, norfenfluramine (norFFA). Adverse events (AEs) were mild to moderate and resolved spontaneously. ZX008 + STP regimen co-administration to healthy adult subjects modestly impacted the number but not severity of AEs. Conclusion: Results show that the STP regimen had a moderate impact on FFA and norFFA PK and ZX008 had no significant impact on the 3 STP regimen drugs. ZX008 would not be expected to alter the clinical response of patients to this regimen by means of an effect on PK. When administering these drugs together, a downward dose adjustment of ZX008 may be warranted.

## Introduction 

Fenfluramine (FFA) has been shown to be effective in open-label studies for the treatment of Dravet syndrome (DS), a severe form of childhood epilepsy also known as severe myoclonic epilepsy of infancy, or SMEI [2]. In patients 1 – 29 years of age with DS, low-dose FFA (0.12 – 1.0 mg/kg/day) has produced prolonged reduction in seizure frequency [3, 4, 5]. Low-dose FFA has also shown preliminary beneficial results in reducing seizure frequency in another form of pharmacoresistant childhood epilepsy, Lennox-Gastaut syndrome (LGS) [6]. 

Most patients with DS or LGS are not adequately controlled by monotherapy, and are treated with multiple antiepileptic drugs (AEDs). Standard AEDs used to treat DS include valproate (VPA), clobazam (CLB), and stiripentol (STP; labeled to be used in combination with CLB and VPA) [7]. Other agents that are currently used in combination therapy for DS include topiramate, levetiracetam [7], felbamate [8], ethosuximide, zonisamide, phenobarbital, clonazepam [2], lamotrigine [9], and cannabidiol [10]. AEDs used to treat LGS include lamotrigine, felbamate, rufinamide, clonazepam, and nitrazepam. These AEDs are believed to treat seizures by altering GABA transmission [11, 12], and some may also act on ion channels [11]. STP also acts by inhibiting cytochrome P450 enzymes, indirectly increasing the concentration of other AEDs metabolized by those enzymes [13]. 

In contrast, FFA pharmacology studies show that it acts on the release and inhibition of reuptake of serotonin [14, 15, 16] and may act directly on select 5-HT receptors [17, 18]. In addition, FFA has been shown more recently to act as a positive allosteric modulator at the σ-1 receptor [19]. Together, these data suggest that the atypical degree and duration of efficacy of FFA observed in open-label studies of the drug in DS [3, 4, 5] and a reduction in seizure frequency in a short-term open-label study in patients with LGS [6] may involve a novel mechanism of action that includes multiple receptors and that is very different from the mechanism of action of other medications currently used to treat these epileptic encephalopathies. 

Polypharmacy in DS and LGS – i.e., the use of multiple AEDs – is often required for seizure reduction, but can lead to increased adverse events (AEs). It is therefore important to characterize the interactions of any new adjunct treatment with currently prescribed AEDs, both to limit additional AEs and to establish proper dosing schedules. The current study assessed the pharmacokinetics (PK) and safety of FFA and its major active metabolite, norfenfluramine (norFFA), following administration of ZX008, an oral solution of FFA (Zogenix, Inc., Emeryville, CA, USA), with and without the co-administration of STP, CLB, and VPA (STP regimen) in healthy subjects. We also examined the PK of STP, CLB, NCLB (nor-clobazam, the major and active metabolite of CLB) [20, 21, 22] and VPA, administered with and without ZX008. 

## Materials and methods 

### Study design and treatments 

This was a phase I, open-label, randomized, single-dose, 3-period crossover study in healthy subjects to evaluate the pharmacokinetic drug-drug interaction between FFA and STP regimen in healthy adult subjects. This study was performed by Quotient Clinical, Ruddington, Nottingham, UK, on behalf of Zogenix International Limited. Study approval was received from the Medicines and Healthcare Products Regulatory Agency and by the appropriate site ethics committee prior to the initiation of the study. The ethics committee that reviewed this study was the Wales Research Ethics Committee 1 located at Castlebridge 4, 15 – 19 Cowbridge Road East, Cardiff, CF119AB, UK. The approval Research Ethics Committee reference number was 16/WA/0010. This study was conducted in accordance with the clinical protocol and with the International Council for Harmonization (ICH) Good Clinical Practice (GCP) Guidelines, including all statutory instruments and amendment regulations. In addition, the study was performed according to the ethical principles outlined in the World Medical Association (WMA) Declaration of Helsinki as currently amended. 

Subjects were screened for inclusion in the study up to 28 days before dosing, and were admitted to the clinical unit on the evening before dosing (day –1). Subjects were randomized on day 1 period 1 to receive one of the three treatments over three study periods, separated by at least a 17-day washout period: A) Single-dose ZX008 0.8 mg/kg; B) STP regimen: STP 3,500 mg + CLB 20 mg + VPA 25 mg/kg (maximum permitted dose of VPA was 1,500 mg, regardless of subject weight); C) ZX008, 0.8 mg/kg + STP regimen. Following an overnight fast of at least 10 hours, each subject received study medication on the morning of day 1 of each of the three treatment periods, based on a randomization code. All subjects then remained fasted until ~ 4 hours post-dosing, when lunch was provided. Water was restricted; when the tablets/capsules of test agents were administered, subjects were given 240 mL of water immediately following oral administration. When subjects were administered VPA as an oral solution from a cup, the dosing vessel was rinsed twice with water and the subject received a total volume, including solution and rinses, of 240 mL. Subjects consumed the rinse solutions immediately after dosing. ZX008 was administered as an oral solution from a dosing syringe and given with 240 mL of water immediately following administration. 

Subjects were confined to the clinical research facility from the evening of day –1 of each treatment period until the morning of day 4 (72 hours after dosing in each treatment period). A follow-up phone call took place on day 17 (± 1 day) of the third treatment period to check on the ongoing well-being of the subjects. 

In each study period, blood samples for PK analysis were obtained, as were blood samples for assessment of clinical laboratory parameters and urine samples for urinalysis. AEs, vital signs, electrocardiogram (ECGs) results, and physical examination findings were noted, and an assessment of suicidality was performed using the Columbia-Suicide Severity Rating Scale (C-SSRS). 

### Subjects 

All subjects gave written informed consent prior to the start of the study. Planned enrollment was 24 healthy subjects. Main inclusion criteria were: males and non-pregnant, non-lactating female subjects; 18 – 50 years old, inclusive; body mass index within the range of 19.0 – 31.0 kg/m^2^; minimum weight of 50.0 kg; and non-smokers for at least 3 months (this included e-cigarettes and nicotine replacement products). Main exclusion criteria included uncontrolled elevated blood pressure; hypersensitivity or idiosyncratic reaction to any of the test agents; taking any medication within 30 days prior to screening that would induce or inhibit hepatic enzymes; Gilbert’s syndrome; positive screen for illicit drugs; history of alcohol abuse; history or presence of a clinically significant medical abnormality or disease; current or past history of cardiovascular or cerebrovascular disease; and a history of renal, hepatic, chronic respiratory, or gastrointestinal disease. 

### Pharmacokinetic evaluation 

Venous blood samples were withdrawn via an indwelling cannula or by venipuncture prior to dosing and at 0.5, 0.75, 1, 1.5, 2, 2.5, 3, 4, 6, 9, 12, 24, 36, 48, and 72 hours following last administration of a study agent. 

For FFA and norFFA, blood samples were collected into 4-mL K_2_EDTA tubes and processed within 30 minutes of collection by centrifugation. A total of 750 µL resultant plasma was transferred into each of two appropriately labeled polypropylene tubes, frozen within 60 minutes of collection and stored at –70 °C or below until analysis of FFA and norFFA using validated analytical methods. The lower limit of quantification was 0.25 ng/mL for both analytes. 

For STP, CLB, NCLB, and VPA, blood samples were collected into a 4-mL lithium heparin tube, immediately stored in ice water, and processed within 45 minutes of collection by centrifugation and decanting of 800 µL of the resultant plasma each into two appropriately labeled polypropylene tubes. Samples were frozen within 45 minutes of collection and were stored at –20 °C or below until analysis of STP, CLB, NCLB, and VPA was completed using validated analytical methods (see below). Lower limit of quantification was 0.25 µg/mL, 25 ng/mL, 3 ng/mL, and 0.5 µg/mL for STP, CLB, NCLB, and VPA, respectively. 

PK was assessed in all subjects who received at least 1 dose of a study agent, who had a minimum of 1 valid post-dose analytical result for PK parameter estimation, and who satisfied the following criteria for at least 1 PK profile: no missing samples at critical time points, no relevant protocol deviations, and no relevant AEs that would suggest insufficient dose absorption. PK parameters for concentrations of FFA and norFFA, STP, CLB, NCLB, and VPA were estimated where possible for each subject and relevant treatment group using non-compartmental analysis methods with Phoenix^®^ WinNonlin^®^ PK software (v6.3, Certara USA, Princeton, NJ, USA). Measured PK parameters included:

- t_max_ (time from dosing to C_max_)
- C_max_ (maximum observed plasma concentration)
- AUC_0–t_ (area under the curve from 0 time to the last measurable concentration)
- AUC_0–inf_ (area under the curve from 0 time to infinity)
- T_1/2_ (terminal elimination half-life)
- CL/F (clearance, the apparent volume cleared of parent drug per unit time after extravascular administration)

### Analytical methods 

FFA and norFFA concentrations in plasma were determined as previously described [23]. Briefly, plasma FFA and norFFA concentrations were quantified by liquid-liquid extraction with methyl t-butyl ether followed by propionic anhydride derivatization and high-performance liquid chromatography (HPLC) with tandem mass spectrometry (MS/MS) detection. Ion transitions monitored were m/z 288.1→159 and 260.1→159 for FFA and norFFA, and m/z 293.1→159 and 266.1→161 for FFA-D_5_ and norFFA-D_6_ (internal standards), respectively. The assay was validated for a range of 0.250 – 100 ng/mL for FFA and norFFA. Plasma concentrations of STP, CLB, NCLB, and VPA were measured by similar methods, with precipitation of plasma proteins with methanol, followed by HPLC with detection by MS/MS, with internal standards of STP-D_9_, CLB-^13^C_6_, NCLB-^13^C_6_, and VPA-D_4_, respectively. Precision and accuracy criteria were ± 15% for all assays. 

### Safety and tolerability 

Safety was assessed in all subjects who received at least 1 dose of an investigational agent throughout the study. Subjects were questioned and/or examined by the investigator or his/her designee for evidence of AEs during screening, prior to dosing and during the 72-hour blood sampling period for each of the three dosing periods. Subjects received a follow-up call on day 17 (± 1 day) following the last blood sampling of the third dosing period to collect AE information. 

### Statistical analysis 

Summary PK values are provided as the mean ± SD, with the exception of the t_max_ summary values, which are presented as the median and range. Statistical analysis was performed for a bioequivalence-type evaluation on log-transformed PK parameters AUC_0–inf_, AUC_0–t_ (also analyzed because AUC_0–inf_ could not be calculated for some subjects due to lack of distinct elimination phase needed to extrapolate AUC to infinity, and used for the calculation of the terminal elimination constant), and C_max_ for each investigational agent unless specified otherwise. To assess the effect of the STP regimen on the PK of FFA and the effect of FFA on the PK of the STP regimen, analyses were performed with SAS (v9.4; SAS Institute, Cary, NC, USA) on the log-transformed AUC and C_max_ data using a mixed effects model with treatment, sequence, and period as fixed effects and subject nested with sequence as a random effect. Adjusted geometric mean ratios (GMRs) and corresponding 90% confidence intervals for the comparisons between ZX008 dosed with and without the STP regimen and for the comparison between the STP regimen dosed with and without ZX008 are provided, where the ratios are defined as ZX008 plus the STP regimen/ZX008 alone and ZX008 plus the STP regimen/STP regimen alone, respectively. p-values (representing the null hypothesis that there is no difference between treatment group means) were calculated for the each of the GMRs. 

## Results 

### Demographics 

Subject demographics are provided in Table 1. A total of 26 subjects enrolled in study. Mean age was 34.5 (range 21 – 50), with a majority of female subjects (n = 15) and Caucasian subjects (n = 23). A majority had history of alcohol consumption, drinking the weekly equivalent of between 0.5 and 3.5 pints (0.2 – 1.7 L) of beer, 1.0 – 6.0 oz (29.6 – 177.4 mL) of a 40% neutral spirit, or 1 – 7 glasses (4.2 oz) (124.2 mL) of wine. There were no notable differences in demographic variables between the different randomization groups. 

A total of 17 subjects completed the study, and 7 withdrew consent due to: inability to attend revised study dates (n = 5), occurrence of an AE (depression, n = 1), and personal reasons (n = 1). In addition, 2 subjects were withdrawn for reasons unrelated to any of the study medications: 1 due to a positive test for drug abuse, and 1 due to an AE requiring a prohibited medication. 

### Pharmacokinetics 

**Figure 1.**
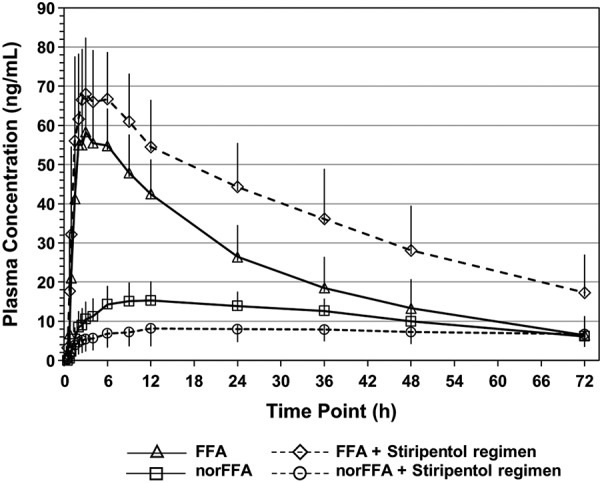
Mean (± SD) plasma concentrations (ng/mL) of fenfluramine (FFA) and norfenfluramine (norFFA) following a single oral dose of ZX008 (0.8 mg/kg) alone and in combination with the stiripentol regimen^a^. ^a^Stiripentol regimen: stiripentol 3,500 mg + clobazam 20 mg + valproate 25 mg/kg (1,500 mg maximum).

Plasma concentration-vs.-time curves for FFA and norFFA in the presence and absence of the STP regimen are provided in Figure 1, while key PK parameters are provided in Table 2. Following a single oral dose of 0.8 mg/kg ZX008 alone, the C_max_ of FFA was 62.4 ± 8.5 ng/mL (values are mean ± SD). When co-administered with the STP regimen, the C_max_ was 75.7 ± 14.7 ng/mL. Thereafter, concentrations declined, remaining quantifiable up to 72 hours post-dose for all subjects and treatments. The mean ± SD terminal half-lives were 20.1 ± 3.3 hours and 22.6 ± 3.8 hours for FFA alone and FFA + the STP regimen, respectively (Table 2). 

NorFFA had a C_max_ of 16.4 ± 4.4 ng/mL when ZX008 was administered alone and 10.2 ± 4.2 ng/mL when administered with the STP regimen. The gradual decline in norFFA plasma concentration over the 72-hour study period resulted in a terminal half-life of 23.5 ± 2.3 hours when ZX008 was administered alone. The plasma half-life of norFFA could not be calculated when ZX008 was administered with the STP regimen because the time frame for half-life calculation was less than 2-fold the calculated half-life for many of the subjects (Table 2). 

Both FFA and norFFA single-dose exposure were significantly altered by the co-administration of the STP regimen (Table 3). FFA C_max_, AUC_0–t_, and AUC_0–inf_ were significantly increased when ZX008 was co-administered with the STP regimen, so that the GMRs of these parameters were statistically greater than 1.0. Conversely, norFFA exposure was reduced when ZX008 was co-administered with the STP regimen, so that C_max_ and AUC_0–t_ GMRs were significantly less than 1.0. 

Table 4 presents key PK parameters for STP, CLB, NCLB, and VPA following administration of the STP regimen alone and in combination with ZX008. Irrespective of whether the STP regimen was dosed in combination with ZX008 or alone, the exposure of STP, CLB, NCLB, and VPA was not altered, as evidenced by the lack of significant difference between the GMRs for C_max_, AUC_0–t_, and AUC_0–inf_ (where available) (Table 5). 

### Safety 

No deaths, severe AEs, or serious AEs (SAEs) were reported. Table 6 presents treatment-emergent AEs (TEAEs) with an incidence ≥ 10% in the overall subject population or in the separate treatment groups. One subject withdrew from the study due to an AE (depressed mood, unrelated to study drug). A second subject took naproxen for pain in the extremity in combination with prophylactic omeprazole, a prohibited medication, and was withdrawn from the study. The majority of subjects reported at least 1 AE; 25 subjects (96.2%) reported a total of 131 AEs. The majority of AEs (110/131) were considered related to the study drugs administered. The highest incidence of AEs was reported following dosing with FFA in combination with the STP regimen (22 [88.0%]) subjects, 64 AEs). A lower incidence of AEs was reported when the STP regimen and ZX008 were administered alone; 16 (76.2%) and 13 (65.0%) subjects reported a total of 32 and 35 AEs, respectively. 

The single administration of these relatively high doses of ZX008, STP, CLB, and VPA in healthy subjects is unlike the clinical treatment, where each drug would be initiated individually and the dose would be gradually titrated to effect. In addition, the ZX008 dose would be capped at 30 mg/day. No subject had a clinically significant clinical laboratory result, vital signs, physical examination, or C-SSRS assessment of suicidality finding. 

## Discussion 

The administration of FFA as ZX008 oral solution did not have any clinically significant impact on the PK of the individual component drugs in the STP regimen, and in fact CLB and VPA were each considered bioequivalent with and without the addition of FFA. We conclude that the addition of ZX008 for a patient taking the STP regimen would not require dose adjustment of STP, VPA, or CLB. The STP regimen did, however, have a significant but moderate impact on the PK of FFA and its metabolite norFFA. Statistically significant increases were observed in the peak plasma levels and systemic exposure (C_max_ and AUC) of FFA when administered with the STP regimen; decreases in C_max_ and AUC for norFFA were observed under these same conditions. These results demonstrate the need to reduce the dose of FFA to account for the PK interaction, when it is administered in combination with the STP regimen. 

PK analysis showed that the co-administration of the STP regimen increased the exposure of FFA by 70% while reducing the exposure of norFFA by 40%. FFA is partially metabolized by CYP1A2, CYP2B6, and CYP2D6, with additional metabolism by CYP2C9, CYP2C19, and CYP3A4. norFFA does not appear to be a strong substrate of any CYP450 enzyme (as discussed below). While the reduction in norFFA exposure appears to be due to a reduction in its formation, there may be a component of reduced elimination of norFFA with the STP regimen that also contributes to this net observed reduction. 

STP is reported to be a strong inhibitor of CYP2C19 and CYP3A4 [13] as well as CYP1A2, CYP2C9, and CYP2D6 [1]. Studies in human liver microsomes conducted by Zogenix confirmed that STP is a time-dependent inhibitor of CYP1A2, CYP2B6, CYP2C9, CYP2C19, and CYP2D6 (Zogenix, data on file). CLB is reported to inhibit CYP2D6, increasing the AUC of the CYP2D6 substrate dextromethorphan by 90% [24]. Because FFA is a substrate for some of these CYP450 enzymes, it appears reasonable that inhibition of these enzymes by the STP regimen could result in increased levels of FFA due to reduced metabolism of FFA to norFFA. The lower levels of norFFA are consistent with reduced formation of norFFA without inhibiting the clearance of norFFA, which indicates norFFA is not a strong substrate for any CYP450 enzyme. 

In addition to metabolism, there is a contribution of renal clearance to the elimination of FFA from the body [25, 26]. Renal elimination of FFA provides a clearance pathway that will not be affected by administration of other drugs that modulate the activity of CYP450 isozymes. Because FFA has multiple pathways of elimination, interference with a single pathway is unlikely to cause a large change in FFA clearance. 

Because many epilepsy patients, and most DS patients, are on more than one antiepileptic medication and some are on as many as five concomitant AEDs [4, 5], it is important to examine the potential PK interactions among AEDs. VPA, CLB, and STP are commonly used therapies for DS, and therefore categorization of any interaction between FFA and these three drugs may be important clinically. 

Subjects in this study were administered active antiepileptic doses of medications that are usually titrated over a period of days to weeks in epilepsy patients. A majority of subjects in the current study reported 1 or more AEs, which were mostly mild in severity with occasional moderate AEs. No significant laboratory findings, vital signs, or C-SSRS assessment of suicidality findings were reported; co-administration of the STP regimen with FFA modestly impacted the number but not the severity of AEs reported. 

## Conclusion 

We conclude from this investigation that FFA had no significant effect on the PK of VPA, STP, CLB, or NCLB, and thus no dose adjustments are needed for these commonly prescribed anticonvulsant medications when administered with ZX008. However, the STP regimen, stiripentol, clobazam, and valproate administered together, had a significant effect on the PK of FFA and norFFA, and thus a downward adjustment of ZX008 dose is recommended when prescribed with the STP regimen. 

## Acknowledgment 

This manuscript was prepared according to the International Society for Medical Publication Professionals’ “Good Publication Practice for Communicating Company-Sponsored Medical Research: The GPP3 Guidelines.” 

## Funding 

This study was funded by Zogenix, Inc. The authors thank Gregory Kopia, PhD, CMPP, and Donald Fallon, ELS, of PharmaWrite, LLC (Princeton, NJ, USA) for medical writing and editorial assistance, which was funded by Zogenix, Inc. 

## Conflict of interest 

BB, AG, BSG, and GMF are employees of, and own stock in, Zogenix, Inc. SS is a consultant to Zogenix and owns no stock. 

**Table 1.** Subject demographics (N = 26).

Baseline characteristic		Male (n = 11)	Female (n = 15)	All (N = 26)
Age, y	Mean ± SD	34.4 ± 9.4	34.7 ± 10.9	34.5 ± 10.1
	(range)	(22 – 47)	(21 – 50)	(21 – 50)
Race, n (%)	White	9 (82)	14 (93)	23 (88)
	Black	2 (18)	0	2 (8)
	Asian	0	0	0
	Other	0	1 (7)	1 (4)
Height, cm	Mean ± SD	179.9 ± 7.5	163.5 ± 5.9	170.5 ± 10.5
	(range)	(167 – 188)	(157 – 180)	(157 – 188)
Weight, kg	Mean ± SD	84.3 ± 14.1	63.9 ± 7.6	72.5 ± 14.8
	(range)	(62.0 – 103.0)	(52.8 – 74.4)	(52.8 – 103.0)
BMI, kg/m^2^	Mean ± SD	25.9 ± 3.0	23.9 ± 2.5	24.7 ± 2.8
	(range)	(22.2 – 30.0)	(21.4 – 29.2)	(21.4 – 30.0)

BMI = body mass index; SD = standard deviation.

**Table 2.** Key pharmacokinetic parameters for fenfluramine (FFA) and norfenfluramine (norFFA) following administration of a single dose of ZX008 (0.8 mg/kg) alone and in combination with the stiripentol (STP) regimen^a^.

Measured agent	n	t_max_ (h)^b^	C_max_ (ng/mL)	AUC_0–t_ (ng×h/mL)	AUC_0–inf_ (ng×h/mL)	T_1/2_ (h)
FFA	19	3.0 (2.0 – 12.0)	62.4 ± 8.5	1,670 ± 497	1,720 ± 491 (n = 17)	20.1 ± 3.3 (n = 17)
FFA + STP regimen	24	3.0 (1.0 – 9.0)	75.7 ± 14.7	2,700 ± 762	2,440 ± 821 (n = 12)	22.6 ± 3.8 (n = 12)
norFFA	19	12.1 (9.0 – 36.0)	16.4 ± 4.4	804 ± 199	839 ± 79 (n = 4)	23.5 ± 2.3 (n = 4)
norFFA + STP regimen	24	24.0 (2.0 – 72.2)	10.2 ± 4.2	520 ± 191	NC	NC

^a^Stiripentol regimen: stiripentol 3,500 mg + clobazam 20 mg + valproate 25 mg/kg (1,500 mg maximum). ^b^t_max_ values are median (range); remaining values are mean ± SD. NC = not calculated. Pharmacokinetic abbreviations are as defined in Materials and methods.

**Table 3.** Statistical analysis of fenfluramine and norfenfluramine following a single dose of ZX008 (0.8 mg/kg) alone and in combination with the stiripentol (STP) regimen^a^.

		Treatment		Statistical comparison		
PK parameter^b^	n	STP regimen + ZX008	ZX008	Ratio (%)	90% CI	p-value
Fenfluramine						
C_max_ (ng/mL)	19	73.7	62.4	118.10	109.43, 127.46	0.002
AUC_0–t_ (ng×h/mL)	19	2,640	1,590	166.19	152.00, 181.71	< 0.001
AUC_0–inf_ (ng×h/mL)	9	2,320	1,370	168.53	154.83, 183.43	< 0.001
Norfenfluramine						
C_max_ (ng/mL)	19	9.11	15.8	57.51	48.80, 67.77	< 0.001
AUC_0–t_ (ng×h/mL)	19	459	782	58.71	50.40, 68.40	< 0.001

^a^Stiripentol regimen: stiripentol 3,500 mg + clobazam 20 mg + valproate 25 mg/kg (1,500 mg maximum). ^b^C_max_ and AUC values are adjusted geometric means. CI = confidence interval; PK = pharmacokinetic. Pharmacokinetic abbreviations are as defined in Materials and methods.

**Table 4.** Key pharmacokinetic parameters for stiripentol, clobazam, norclobazam, and valproate following administration alone (stiripentol regimen^a^) and in combination with ZX008 (0.8 mg/kg).

Measured agent in combination	n	t_max_ (h)^b^	C_max_ (ng/mL)	AUC_0–t_ (ng×h/mL)	T_1/2_ (h)
Stiripentol	19	6.0 (1.1 – 48.1)	3,280 ± 1,390	81,800 ± 31,100 (n = 17)	16.0 ± 4.2 (n = 9)
Stiripentol + ZX008	24	6.0 (1.5 – 36.0)	3,690 ± 1,210	76,900 ± 33,800	16.7 ± 7.5 (n = 12)
Clobazam	19	3.0 (1.5 – 6.0)	231 ± 38.9	7,960 ± 1,120 (n = 17)	28.9 (n = 1)
Clobazam + ZX008	24	3.0 (1.5 – 6.0)	216 ± 54.2	7,750 ± 1,600	25.6 ± 2.1 (n = 4)
Norclobazam	19	72.0 (48.0 – 72.2)	97.4 ± 30.9	4,380 ± 1,500 (n = 17)	NC
Norclobazam + ZX008	24	72.0 (71.6 – 72.2)	94.8 ± 28.5	4,420 ± 1,570	NC
Valproate	20	0.8 (0.5 – 3.0) (n = 18)	100,000 ± 9,140 (n = 19)	1,720,000 ± 415,000 (n = 17)	15.0 ± 2.9 (n = 17)
Valproate + ZX008	24	1.0 (0.5 – 3.0)	107,000 ± 17,600	1,890,000 ± 402,000	14.8 ± 2.5

^a^Stiripentol regimen: stiripentol 3,500 mg + clobazam 20 mg + valproate 25 mg/kg (1,500 mg maximum). ^b^t_max_ values are median (range); remaining values are mean ± SD, except where SD is not calculated. NC = not calculated. Pharmacokinetic abbreviations are as defined in Materials and methods.

**Table 5.** Statistical analysis of stiripentol, clobazam, norclobazam, and valproate following administration of the stiripentol (STP) regimen^a^ alone and in combination with ZX008 (0.8 mg/kg).

Measured AED^b^		Treatment		Statistical comparison		
	n	STP regimen	STP regimen + ZX008	Ratio (%)	90% CI (%)	p-value
Stiripentol						
C_max_ (ng/mL)	18	3,030	3,500	115.6	97.9 – 136.5	0.15
AUC_0–t_ (ng×h/mL)	16	85,500	74,200	86.7	68.0 – 110.6	0.33
Clobazam						
C_max_ (ng/mL)	18	241	229	95.3	88.0 – 103.2	0.30
AUC_0–t_ (ng×h/mL)	16	8,130	7,710	94.8	90.3 – 99.6	0.079
Norclobazam						
C_max_ (ng/mL)	18	99.3	99.8	100.5	96.5 – 104.8	0.82
AUC_0–t_ (ng×h/mL)	16	4,410	4,480	101.6	95.0 – 108.6	0.68
Valproate						
C_max_ (ng/mL)	18	104,000	105,000	101.3	96.6 – 106.2	0.64
AUC_0–t_ (ng×h/mL)	16	1,760,000	1,750,000	99.6	94.1 – 105.3	0.89

^a^Stiripentol regimen: stiripentol 3,500 mg + clobazam 20 mg + valproate 25 mg/kg (1,500 mg maximum). ^b^C_max_ and AUC values are adjusted geometric means. AED = antiepileptic drug; CI = confidence interval. Pharmacokinetic abbreviations are as defined in Materials and methods.

**Table 6.** Incidence of TEAEs with a frequency of ≥ 10% for ZX008 alone, stiripentol (STP) regimen alone, and ZX008 + STP regimen groups.

System/organ class	ZX008 (n = 20) n (%)	STP regimen (n = 21) n (%)	ZX008 + STP regimen (n = 25) n (%)	Overall (n = 26) n (%)
Subjects reporting TEAEs	13 (65.0)	16 (76.2)	22 (88.0)	25 (96.2)
Nervous system disorders	9 (45.0)	14 (66.7)	20 (80.0)	24 (92.3)
Somnolence	1 (5.0)	12 (57.1)	19 (76.0)	20 (76.9)
Headache	6 (30.0)	3 (14.3)	7 (28.0)	13 (50.0)
Dizziness	3 (15.0)	1 (4.8)	2 (8.0)	5 (19.2)
Gastrointestinal disorders	6 (30.0)	4 (19.0)	9 (36.0)	14 (53.8)
Nausea	2 (10.0)	1 (4.8)	7 (28.0)	10 (38.5)
Vomiting	1 (5.0)	2 (9.5)	4 (16.0)	6 (23.1)
General disorders and administration site conditions	5 (25.0)	2 (9.5)	4 (16.0)	11 (42.3)
Fatigue	3 (15.0)	2 (9.5)	2 (8.0)	7 (26.9)
Psychiatric disorders	2 (10.0)	1 (4.8)	5 (20.0)	7 (26.9)
Anxiety	2 (10.0)	0	2 (8.0)	3 (11.5)
Metabolism and nutrition disorders	2 (10.0)	0	3 (12.0)	3 (11.5)
Decreased appetite	2 (10.0)	0	3 (12.0)	3 (11.5)

TEAEs = treatment-emergent adverse events.
